# PRESSURED TOUCH FOOT CARE TO RELIEVE CHRONIC PAIN AND IMPROVE QUALITY OF LIFE

**DOI:** 10.1093/geroni/igad104.3533

**Published:** 2023-12-21

**Authors:** Mary Clayton-Jones

**Affiliations:** University of Massachusetts, Amherst/ FootCare by Nurses, Amherst, Massachusetts, United States

## Abstract

**Background:**

Chronic pain affects many older adults and is a leading cause of disability. It is often treated pharmacologically, but evidence indicates touch therapy modalities are beneficial. Purpose: To implement a therapeutic touch program for adults enrolled in a Program of All-Inclusive Care for the Elderly (PACE) to help reduce chronic pain and improve quality of life for those patients enrolled in this project.

**Method:**

Twelve participants in an assisted living facility with chronic foot pain were initially selected by the PACE Family Nurse Practitioner (FNP) for home visits with this Doctor of Nursing Practice (DNP) student for a foot care and pressured touch intervention to occur once a month for 3 months. The McGill Pain and Quality of Life Scores questionnaires were administered before each intervention.

**Results:**

Four participants completed all three interventions. Every participant experienced a decrease in pain pre- and post-intervention and month-to-month of 15% or more. Three participants had experiences of complicated grief and had a decrease in quality-of-life scores over the 3 months. The participant without complicated grief had an improvement in the quality-of-life scores with pain reduction.

**Conclusions:**

This small home intervention project for chronic foot pain demonstrated that foot care and pressured touch may be a cost-effective adjunct therapy for reducing pain. Chronic pain can be complicated by grief, which must be considered when looking to support elder wellness and quality of life. Keywords: Chronic pain, therapeutic touch

